# TINCR inhibits the proliferation and invasion of laryngeal squamous cell carcinoma by regulating miR-210/BTG2

**DOI:** 10.1186/s12885-021-08513-0

**Published:** 2021-06-29

**Authors:** Guoqing He, Rui Pang, Jihua Han, Jinliang Jia, Zhaoming Ding, Wen Bi, Jiawei Yu, Lili Chen, Jiewu Zhang, Yanan Sun

**Affiliations:** 1 Department of Otorhinolaryngology, Head and Neck Surgery, The Second Affiliated Hospital, Harbin Medical University, No. 246 Xuefu Road, Harbin, 150086 China; 2 Department of Head and Neck Surgery, Harbin Medical University Cancer Hospital, No.150 Haping Road, Harbin, 150081 China

**Keywords:** Laryngeal squamous cell carcinoma, TINCR, miR-210, TU212, BTG2

## Abstract

**Background:**

Terminal differentiation-induced ncRNA (TINCR) plays an essential role in epidermal differentiation and is involved in the development of various cancers.

**Methods:**

qPCR was used to detect the expression level of TINCR in tissues and cell lines of laryngeal squamous cell carcinoma (LSCC). The potential targets of TINCR were predicted by the bioinformation website. The expression of miR-210 and BTG2 genes were detected by qPCR, and the protein levels of BTG2 and Ki-67 were evaluated by western blot. CCK-8 assay, scratch test, and transwell chamber were used to evaluate the proliferation, invasion, and metastasis ability of LSCC cells. The relationships among TINCR, miR-210, and BTG2 were investigated by bioinformatics software and luciferase reporter assay. The in vivo function of TINCR was accessed on survival rate and tumor growth in nude mice.

**Results:**

We used qRT-PCR to detect the expression of TINCR in laryngeal squamous cell carcinoma (LSCC) tissues and cells and found significantly lower levels in cancer tissues compared with adjacent tissues. Additionally, patients with high TINCR expression had a better prognosis. TINCR overexpression was observed to inhibit the proliferation and invasion of LSCC cells. TINCR was shown to exert its antiproliferation and invasion effects by adsorbing miR-210, which significantly promoted the proliferation and invasion of laryngeal squamous cells. Overexpression of miR-210 was determined to reverse the tumour-suppressive effects of TINCR. BTG2 (anti-proliferation factor 2) was identified as the target gene of miR-210, and BTG2 overexpression inhibited the proliferation and invasion of LSCC cells. BTG2 knockdown relieved the inhibitory effects of TINCR on the proliferation and invasion of LSCC. Finally, TINCR upregulation slowed xenograft tumour growth in nude mice and significantly increased survival compared with control mice.

**Conclusion:**

The results of this study suggest that TINCR inhibits the proliferation and invasion of LSCC by regulating the miR-210/BTG2 pathway, participates in cell cycle regulation, and may become a target for the treatment of LSCC.

**Supplementary Information:**

The online version contains supplementary material available at 10.1186/s12885-021-08513-0.

## Background

Laryngeal cancer is an aggressive head and neck tumour. The most common pathological subtype (96 to 98%) is laryngeal squamous cell carcinoma (LSCC) followed by adenocarcinoma, basal cell carcinoma, poorly differentiated carcinoma, and sarcoma. Laryngeal cancer is one of the most common malignant tumours of the head and neck [1]. The initiation and development of laryngeal cancer is a biological process involving multiple factors. With the completion of the human genome sequence, researchers have found that in addition to traditional genes, the genome is composed of more than 90% “dark matter”, which includes non-coding RNAs (ncRNAs), of which long noncoding RNAs (lncRNAs) were recently shown to be involved in the occurrence and progression of laryngeal cancer [2, 3].

lncRNA is an RNA transcript longer than 200 nucleotides. Most lncRNAs cannot encode proteins but have rich biological functions. A small portion of lncRNAs can also encode polypeptides. lncRNAs can fold into complex secondary/tertiary structures and scaffolds that interact with various proteins, transcription regulators, mRNAs (complementary), and DNA sequences [4, 5]. In the progression of human LSCC, abnormal lncRNA expression is often closely associated with prognosis. For example, Qu et al. found that abnormally high expression of hoxa11-as is often seen in LSCC tissues, and patients with high hoxa11-as expression tend to have a poor prognosis [6].

We analysed some lncRNAs in LSCC tissue samples and found multiple abnormally expressed lncRNAs, including TINCR, HOTAIR, NEAT1, LINC00520, AC016747.3, miR-155HG, and H19 (Supplemental Material 1, Fig S1B). Among these, TINCR aroused our interest. TINCR was first identified by Kretz et al., who showed that TINCR was induced in the late differentiation phase of epidermal tissue. The size of TINCR is approximately 3.7 kb, and its function is closely related to cell differentiation [7]. Recently, evidence has accumulated showing that TINCR can actively participate in cancer progression [8]. For example, TINCR can regulate cell proliferation, migration, and invasion by potentially targeting CLND7 and ANAX1 to induce ESCC [9]. TINCR regulates the expression of PDK1 by sponging miR-375, thereby promoting the development of gastric cancer by inhibiting apoptosis and promoting cell proliferation [8]. Based on the above important role of TINCR in other cancers, we speculated that TINCR might affect the progression of LSCC.

MicroRNAs (miRNAs) are endogenous, evolutionarily conserved ncRNAs with a length of 21–23 nt [5]. miRNAs act as oncogenes or tumour suppressor genes by regulating many biological events, such as cell differentiation, proliferation, and apoptosis [10, 11]. Studies have shown that lncRNAs can be used as miRNA-competitive endogenous RNAs (ceRNAs) regulating the expression of corresponding targeted miRNAs [12–15]. The abnormal expression of lncRNAs leads to disorder of the lncRNA-miRNA-mRNA network and interrupts a steady state of gene expression, which may cause cancer development. Therefore, this study aims to determine the role of TINCR in the development of laryngeal carcinoma and explore its interaction with other miRNAs to elucidate the underlying mechanism.

## Methods

### HNSCC tissue sample material

The study is compliant with all relevant ethical regulations for human research participants. All participants provided written informed consent. Forty-five cases of laryngeal carcinomas and laryngopharyngeal carcinomas were surgically removed and collected from the Third Hospital of Harbin Medical University. Each specimen was excised from cancer tissues and matched with adjacent tissues. The tumour edges were evenly cut within 20 min, avoiding necrotic tissue. The total volume was approximately 0.2–0.5 cm^3^, and samples were deposited into labelled cryotubes. Each specimen was immediately stored at − 80 °C for protein and RNA extraction and subsequent Western blots and qRT-PCR. The Harbin Medical University Ethics Committee supported the research.

### Cell lines

The human LSCC cell line Hep-2 was obtained from the Shanghai Cell Bank of the Chinese Academy of Sciences, and TU212, TU686, M2e, and M4e cells were obtained from Zhejiang Ruyao Biotechnology Co., Ltd. Cells were cultured in Dulbecco’s modified Eagle’s medium (DMEM) containing 10% foetal bovine serum and were grown in a 37 °C cell culture incubator containing 5% CO_2_.

### Laboratory animals

In this study, 4- to 5-week-old male BALB/C nude mice (SPF grade, *n* = 16) were purchased from Shanghai SLAC Laboratory Animal Co., Ltd. and reared in the IVC barrier system. At the end of experiments, the mice were euthanized by CO_2_ asphyxiation. Animal experiments were performed following the standards in Harbin Medical University’s Regulations for the Management of Laboratory Animals and were approved by the Harbin Medical University Laboratory Animal Ethics Committee.

### Lentivirus transduction

The plasmids pCDH-TINCR and pCDH-BTG2 for the lentivirus package were constructed by Zhejiang Ruyao BioTechnology Co., Ltd. Lipofectamine® 3000 transfection reagent and puromycin were purchased from Thermo Fisher Scientific, Inc. (Waltham, MA, USA). TU212 human laryngeal cancer cells were cultured in RPMI-1640 with 10% foetal bovine serum. Twenty-four hours before transfection, 293 T cells were trypsinized and divided them into 6-cm plates. Cells were transfected at 70–80% confluence, and the number of cells transfected by lentivirus was approximately 2 × 10^5^/well. The next day, 2 mL fresh medium containing 6 μg/mL polybrene was used to replace the original medium. Fifty microlitres of concentrated virus suspension was added and incubated at 37 °C. After 4 h, 2 mL fresh medium was added to dilute polybrene, and the culture was continued for 24 h. The medium containing virus was replaced with fresh medium. After culture for 3 days, 2 μg/mL puromycin was added for screening. Because the lentivirus conferred fluorescent protein expression, fluorescence was visualized 48 or 72 h after transfection to indicate successful transfection. PCR primers for amplification were as follows: TINCR-Nhe I-F, ATC TTA ATG CTA GCG GGC GGG CGG AGC GCG GGC G; TINCR-BstB I-R, GGA CCA TTT CGA ATT GTT TTC AAA CAT GTA ATC T; BTG2-BamHI-F, 5`-CGG ATC CAG CAC TAC AAA CAC CAC TG-3`; BTG2-NheI-F, 5`-CTA GCT AGC CCC TTC CCG TGG CTC ATA A-3`. Lentiviruses for miR-210, miR-con, anti-miR-210, and anti-miR-con were provided by Genewiz Biotechnology Co., Ltd. (Jiangsu, China).

### Transfection

siRNA targeting TINCR (si-TINCR) was synthesized by GenePharma Co. LTD (Shanghai, China). siRNA and lentivirus containing green fluorescent protein (GFP) were constructed by GeneChem (Shanghai, China). siRNA sequences targeting TINCR were as follows: 5′-GUG AAG UCU UAG AAA CUU UCC-3′ (siRNA-1) and 5′-UUA ACU AAA ACA UUA UUU CUU-3′ (siRNA-2). siRNA sequences targeting BTG2 were as follows: 5′-UCA UUU AAA AAU ACA GUU CCC-3′ (siRNA-1) and 5′-UCU UCA UUU AAA AAU ACA GUU-3′ (siRNA-2).

### Cell proliferation test

A CCK-8 kit (Cell Counting-8 kit) was used to detect cell proliferation. The cells were plated in 96-well plates at a density of 2000 cells per well with three replicate wells per group. Cells were placed in a 37 °C incubator, and at time intervals of 24 h, 48 h, and 72 h, 10 μL of CCK8 reagent was added to each well and incubated for another 4 h in the 37 °C 5% CO_2_ incubator. The optical density (OD) of each well was measured with a microplate reader at a wavelength of 450 nm, and each group’s proliferative capacity was calculated.

### Scratch test

Cells were seeded in a 6-well plate, grown to approximately 90% confluence, and a line was scratched through the single layer of cells using a 10-μL plastic tip. After washing three times with PBS to remove debris, the cells were cultured with fresh DMEM containing 5% FBS. Wound healing of the scratch line after 24 h was observed. The images were photographed under an inverted microscope (DMIL LED, Leica) at 100× magnification. The cell scratch width of the acquired images was measured by IPP 6.0 software. Each experiment was performed in triplicate in at least two independent experiments.

### Transwell

Forty-eight hours after cell transfection, cells were resuspended by trypsin digestion. The cell density was adjusted to 5.0 × 10^4^ cells/mL, 200 μL of the cell suspension was inoculated into the upper chamber of the Transwell plate, and 600 μL of the complete medium was added to the lower chamber. Then, the chamber was incubated in a 37 °C CO_2_ incubator for 24 h. The chamber was removed and fixed with paraformaldehyde. Cells were stained with haematoxylin for 15 min and observed with an inverted microscope (DMIL LED, Leica) at 400× magnification. Five fields were randomly selected to count the number of transmembrane cells.

### Cell cycle analysis

Collected cells were fixed with precooled ethanol at 4 °C for 1 h, and then the cells were fixed and washed with prechilled PBS and resuspended in 1 mL PBS containing 50 μg/mL RNase and 50 μg/mL PI staining solution. After incubation at 37 °C for 20 min, the cell DNA content was analysed by flow cytometry. Each sample was guaranteed to have 20,000 cells, and the cell cycle analysis software FlowJo was used to determine the cell cycle distribution and proportions.

### Xenograft model

The animals used in this experiment were 4-week-old BALB/C athymic nude mice(*n* = 16). All mice were injected subcutaneously in the dorsal scapular region with 100 μL of a suspension (1 × 10^6^) of TU212 cells. The size and volume of the tumours were measured every 2 days using callipers twice a week using a simplified formula of a spheroid (0.5× length × width^2^ = 0.5 × a × b^2^, mm^3^). Once the tumour reached approximately 0.5–0.6 cm^3^, the mice received an injection once a week for three weeks. The experimental group (*n* = 8) mice received 100 μL pCDH-TINCR lentiviral treatment, whereas the control group (n = 8) mice received 100 μL lentiviral GFP injection. Nude mice were sacrificed 1 week after the end of treatment to obtain tumours.

### Quantitative real-time PCR analysis

Total RNA was extracted using TRIzol. Two micrograms of total RNA were reverse transcribed into cDNA using a high-capacity cDNA reverse transcription kit. Quantitative PCR used Power SYBR Green RT-PCR reagent. The primers were as follows: TINCR-qPCR-F, 5′-CTT CCC TTT AAT ATC CAT TCA C-3′; TINCR-qPCR-R, 5′-CTC TTC CCA CAT ACA AAC ATA CAT ACA TAC-3′; miR-210-qPCR-F, 5′- TGT GCG TGT GAC AGC G-3′; and miR-210-qPCR-R, 5′-GAA CAT GTC TGC GTA TCT C-3′. All reactions were performed on a 7500F real-time PCR system. The mRNA and lncRNA gene expression levels were normalized using GAPDH as the internal reference gene and then calculated with the 2^-ΔΔct^ method.

### Western blots

Cells were collected and a protein lysate was prepared (PMSF:RIPA = 1:100). An appropriate lysis buffer volume was added to cells and then incubated on ice for 30 min. Lysate was centrifuged at 12,000 g for 30 min at 4 °C, and the supernatants were collected. The concentration of total protein was measured using a bicinchoninic acid (BCA) assay kit (Boster, Wuhan, China). After adding 5× loading buffer, the protein sample was boiled for 5 min. Denatured protein was added to a 10% SDS-PAGE gel for electrophoretic separation at 80 V and 150 mA. Protein was then transferred to a membrane for 100 min. Proteins were blocked with 5% skim milk for 2 h. The relevant antibody was diluted with a primary antibody dilution solution at a specific ratio (rabbit anti-GAPDH as an internal reference, dilution ratio of 1:1000, #5174, CST, USA). The primary antibodies rabbit anti-Ki67 (#9449, 1:1000, CST, USA) and BTG2 (ab273659, 1:1000, Abcam, UK) were added separately and incubated at 4 °C overnight. The next day (after 12–16 h), the cells were washed with TBST 3 times for 10 min each. Then, the corresponding HRP-conjugated goat anti-rabbit secondary antibody (#7074, diluted 1:2000, CST, USA) was added and incubated at room temperature on a shaker for 2 h, and secondary antibodies were detected by chemiluminescence. Protein levels were analysed using ImageJ.

### HE staining

Tumour tissues fixed with formalin for more than 48 h were removed, trimmed for dehydration and clearing, and dipped in wax. The wax-soaked tissue was placed in an embedding machine, embedded in a trimmed wax block on a microtome, and placed in a 60 °C oven to melt the paraffin. Haematoxylin-eosin staining was then performed. Alcohol gradient dehydration was used, xylene was used to make transparent neutral gum, and the sections were covered with glass to seal. After completion, the slides were observed under an optical microscope (Leica, DM500), the magnification was 100× and 400×, the pictures were taken by Oplenic DIGITAL CAMERA, and the images were collected by the Oplenic system, and the tumour tissue area was quantified to determine the degree of cancer pathology.

### Immunohistochemistry

The tissue was baked in a 60 °C incubator for 20 min, dewaxed twice with xylene, soaked with anhydrous ethanol 2 times for 5 min each, and then soaked in 95% ethanol, 80% ethanol, and 70% ethanol for 2 min each. After washing with PBS, the tissue was incubated with a closed permeable solution containing 3% H_2_O_2_ for 10 min. The slices were placed in 0.01 M citric acid buffer (pH 6.0), and the antigen was repaired in a 121 °C autoclave for 20 min. Then, 5% goat serum blocking solution was added and incubated at room temperature for 20 min. Ki67 antibody was diluted at a ratio of 1:100; 50 μL antibody was added to the covering tissue, and the sample was incubated at 4 °C overnight. The cells were washed with PBS 2–3 times for 5 min each. HRP-labelled sheep anti-rabbit secondary antibody was added and incubated at room temperature for 1 h. Then, DAB colour was developed, haematoxylin was re-dyed, alcohol gradient dehydration was performed, xylene was transparently treated, neutral gum was sealed, and samples were observed and photographed under a microscope (The image shooting equipment and operation refer to HE dyeing).

### Bioinformatics analysis

The bioinformatics websites miRDB (http://www.mirdb.org) and LncRNA2 Target v2.0 were used to predict the potential binding sites of lncRNAs to miRNAs. TargetScan (http://www.targetscan.org), miRDB (http://mirdb.org), and miRanda (http://www.microrna.org) were used to predict the gene targets of miRNAs and binding sequences.

### Luciferase report analysis

Partial sequences of TINCR and BTG2 (anti-proliferation factor 2) 3’UTRs containing wild-type and mutant miR-210 binding sites were cloned into pGL3-Basic luciferase vector (Promega, Madison, WI, USA) to generate TINCR-WT, TINCR-MUT, BTG2–3′ UTR-WT (WT), and BTG2–3′ UTR-MUT (MUT). The constructed luciferase vector was then transfected into laryngeal squamous carcinoma cells with the pRL-TK vector (Promega) and miR-con, miR-210, anti-miR-con, or anti-miR-210. A dual-luciferase reporter gene detection system (Promega) was used to detect the luciferase activity of the cell lysate 48 h after transfection. The primers were as follows: TINCR-qPCR-F, 5′-CTT CCC TTT AAT ATC CAT TCA C-3′; TINCR-qPCR-R, 5′-CTC TTC CCA CAT ACA AAC ATA CAT ACA TAC-3′; miR-210-qPCR-F, 5′- TGT GCG TGT GAC AGC G-3′; and miR-210-qPCR-R, 5′-GAA CAT GTC TGC GTA TCT C-3′.

### Statistical analysis

All the above experiments were repeated 3 times separately, and the data are expressed as the means and standard deviations. The statistical software SPSS 19.0 was used between multiple groups of data to analyse differences using one-way analysis of variance (ANOVA). A *P*-value < 0.05 was considered statistically significant.

## Results

### Downregulation of TINCR expression in LSCC

**Fig. 1:**
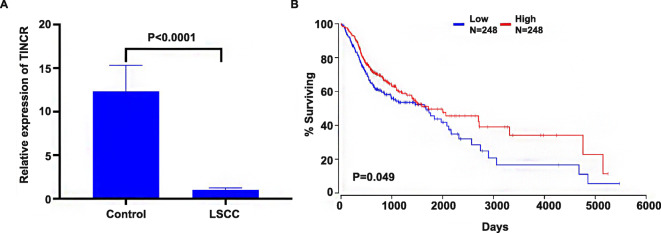
The relationship between TINCR expression level and prognosis in laryngeal carcinoma. (**A**) The gene expression level of TINCR in adjacent tissues (control group) and laryngeal cancer tissues (LSCC group). (**B**) Relationship between different TINCR expression levels and survival rate in patients with LSCC

Gene expression in tissue samples from LSCC patients was detected by RT-PCR. TINCR expression was significantly decreased in LSCC tissues compared with adjacent tissues (Fig. 1A). LSCC patients with low TINCR expression showed a significantly worse prognosis (*P* < 0.05, Fig. 1B).

### Construction of stable TINCR-overexpressing cells

**Fig. 2:**
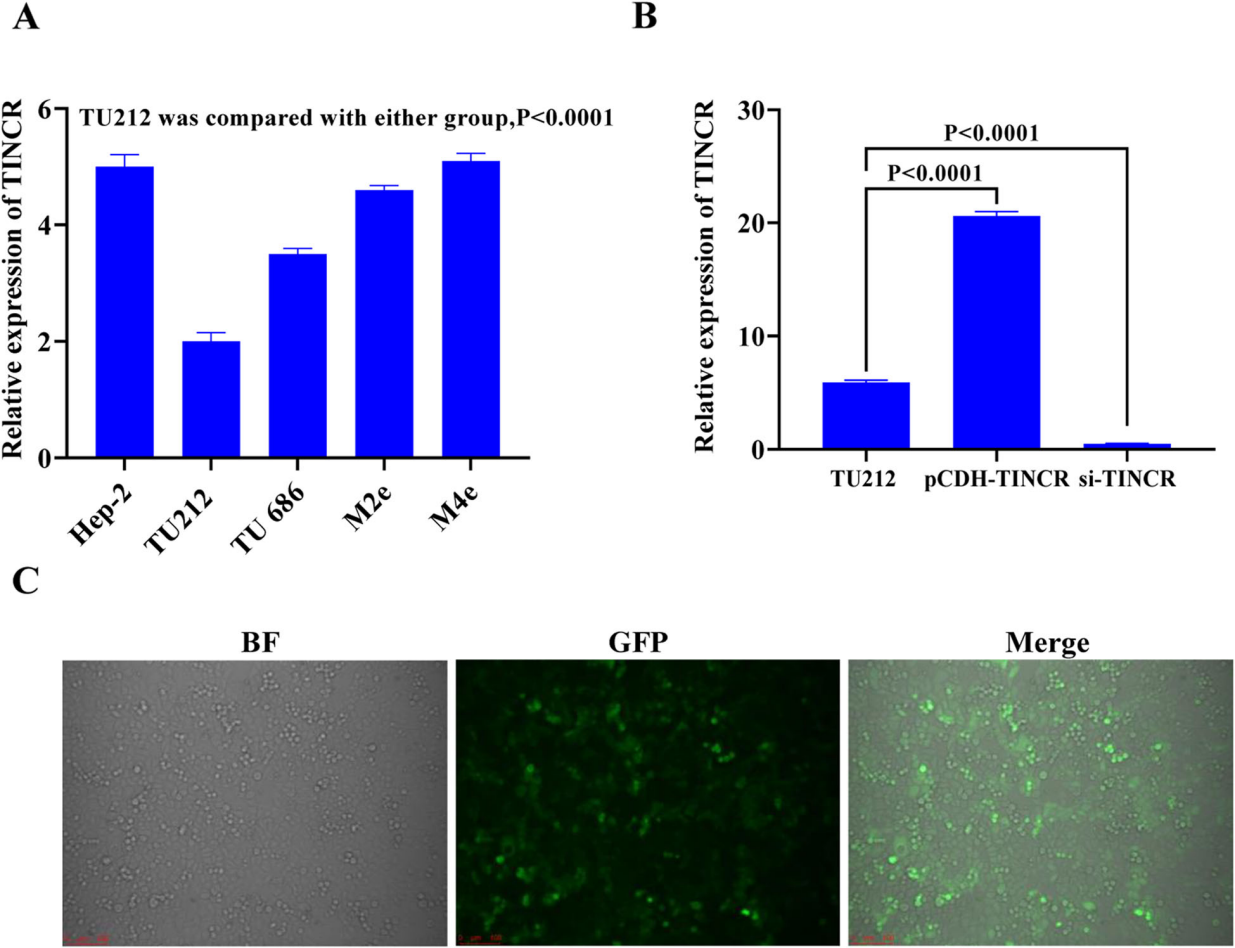
The effect of TINCR overexpression and silencing on the proliferation rate of laryngeal cancer cells. (**A**) qPCR was used to detect the relative expression of the TINCR gene in different laryngeal cancer cell lines. ** indicates that there was a significant difference between the control group and different laryngeal cancer cell lines (*P <* 0.01). (**B**) Virus infection or siRNA transfection of TU212 cells. The expression level of TINCR was detected by qPCR. (**C**) Fluorescence diagram of TU212 cells 48 h after TINCR lentivirus infection. Images include bright field, green fluorescence, and superimposition of the two. The resolution is 72 dpi

We performed RT-PCR to detect TINCR in different laryngeal cancer cell lines. As shown in Fig. 2A, the expression of TINCR was the lowest in TU212 cells. At the same time, we combined Transwell and CCK-8 to detect the invasion and proliferation of these cell lines. Transwell results showed that TU212 had the most vital invasion ability, but found no significant difference in proliferation ability between the groups in the detection of CCK-8(Supplementary material 1, Fig. S1C and S1D). Therefore, we chose TU212 as the primary research cell line for subsequent experiments. Lentiviral transfection was used to infect TU212 cells (Fig. 2C), and qPCR detection demonstrated successful TINCR overexpression. Transfected siRNA was used to inhibit TINCR expression, and fluorescent quantitative PCR also verified successful TINCR expression inhibition (Fig. 2B).

### TINCR inhibits the proliferation and invasion of TU212 cells

**Fig. 3:**
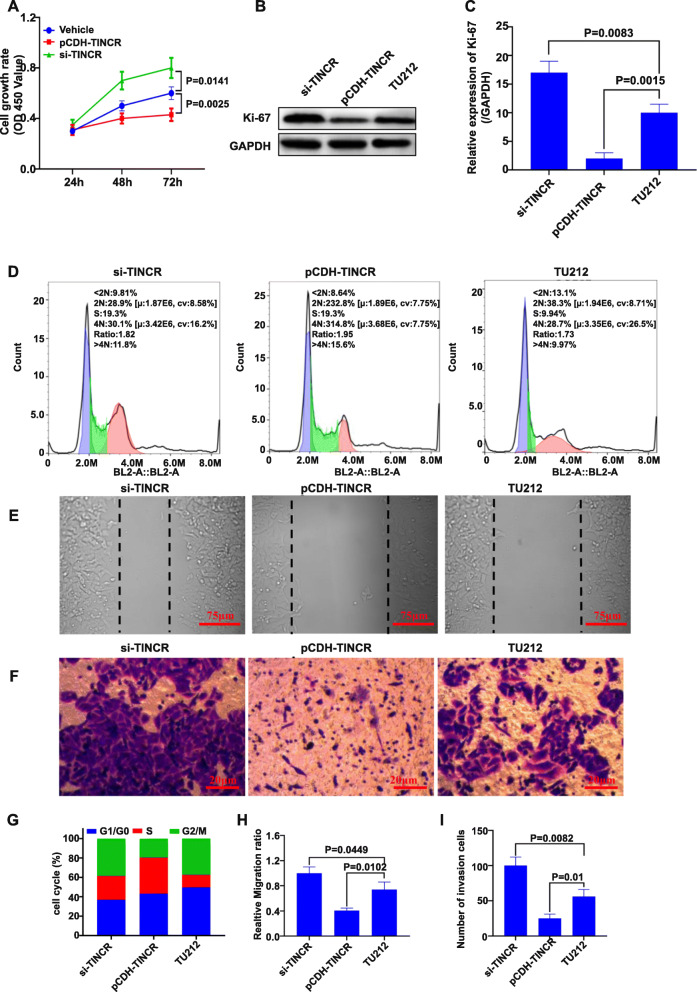
The effect of TINCR overexpression and silencing on laryngeal cancer cell proliferation. (**A**) The proliferation rate measured by CCK-8, which is expressed as OD 450 nm absorbance. (**B**) The expression of Ki67 protein, a proliferation-related protein detected by WB. (**C**) Quantification of Western blot results in B. (**D**) Cell cycle measured by flow cytometry. (**E**) Cell scratches. (**F**) Transwell assay of the invasion of silenced or overexpressed TINCR cells. (**G**) Statistical diagram of cell cycle proportions o. (**H**) Quantification of relative cell migration rates. (**I**) Quantified graph of Transwell experiment results. The resolution is 72 dpi

CCK-8 test results showed that cell proliferation was inhibited in the TINCR overexpression group, and the difference was statistically significant (*P <* 0.05). Silencing TINCR increased the cell proliferation rate (Fig. 3A). To further confirm the effect of TINCR on cell proliferation, Western blotting was used to detect the expression level of Ki67, a cell marker associated with proliferation in different groups (Fig. 3B and C). The overexpression of TINCR in TU212 cells significantly inhibited the expression of Ki67 (*P <* 0.05). After TINCR was silenced, Ki67 protein expression was significantly increased. This result confirmed that TINCR could inhibit the proliferation of laryngeal cancer cells. Analysis of the cell cycle by flow cytometry (Fig. 3D and G) showed that TU212 cells with virus-induced TINCR upregulation were arrested in the S phase 72 h after transduction. The ratio increased significantly (*P <* 0.05) compared with other groups, and the ratio of G2/M, which represents cell division, was significantly decreased. However, compared to TU212 cells after TINCR knockout, the proportion of cells remaining in the G1 phase was significantly increased. This result suggested that TINCR inhibited the proliferation of LSCC cells by affecting the cell cycle. The cell scratch test was then used to evaluate cell migration. Scratch healing was observed after 24 h (Fig. 3E). After TINCR inhibition, the migration of TU212 cells was improved. Cells migrated into the scratched region after 24 h. However, after overexpressing TINCR, migration was suppressed, which was reflected in the broader scratches. The results suggested that TINCR overexpression significantly inhibited the migration of TU212 laryngeal cancer cells. The difference was statistically significant compared with the parental cells or with the TINCR-silenced group (Fig. 3H, *P <* 0.05). We also tested the ability of TINCR to affect TU212 cell invasion in Transwell assays. The results showed that with TINCR overexpression, as shown in Fig. 3F and I, the invasion of TU212 cells was significantly inhibited, and the downregulation of TINCR significantly promoted the invasive capacity of TU212 cells.

### TINCR adsorbs miR-210

**Fig. 4:**
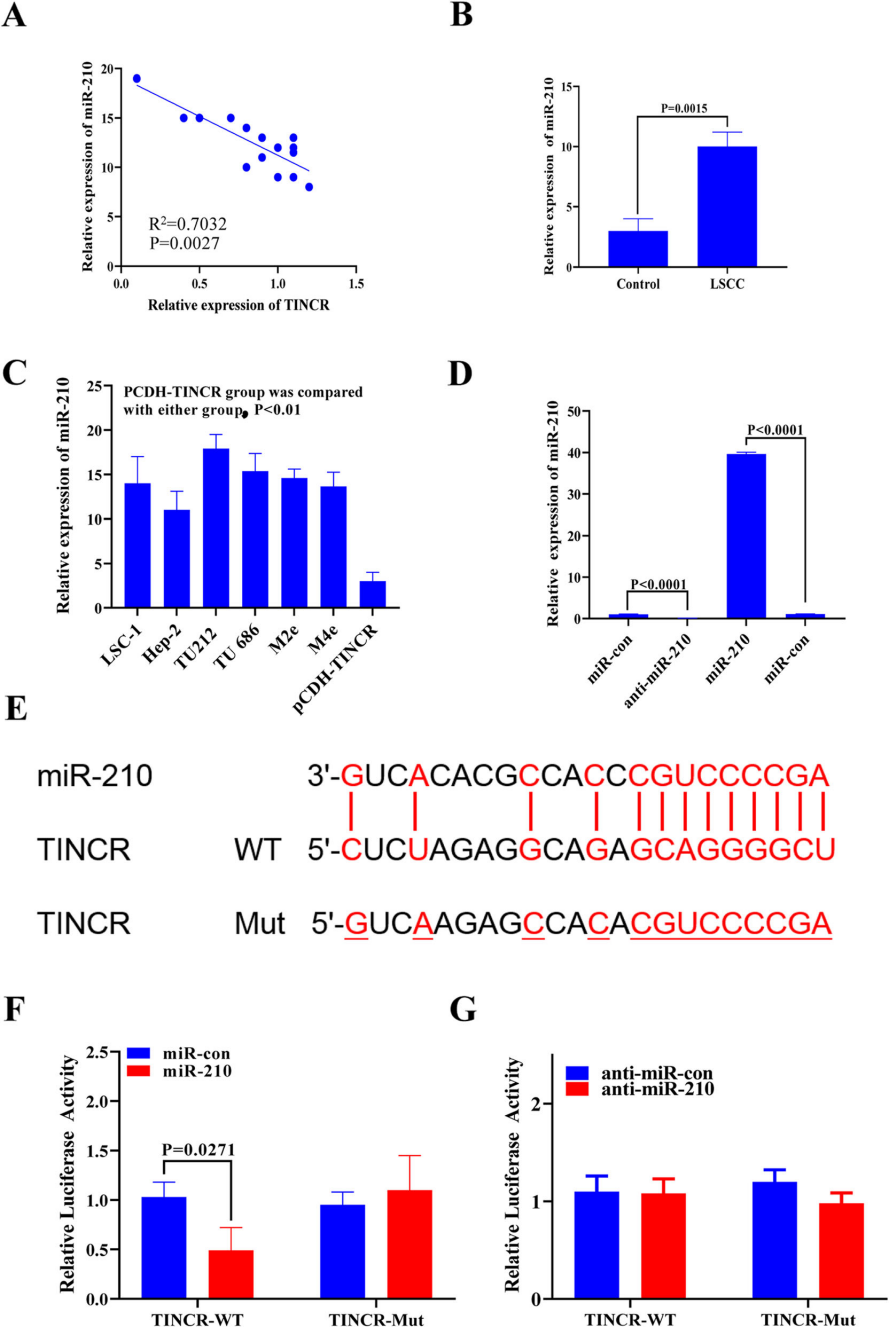
TINCR adsorption of miR-210. (**A**) Through sponge adsorption, the expression level of the TINCR gene and miR-210 gene in LSCC tissues showed a linear, negatively correlated relationship, R^2^ = 0.7032. (**B**) The expression of miR-210 in laryngeal cancer tissues and adjacent tissues. (**C**) qPCR detection of miR-210 in cells. (**D**) miR-210 expression detected by qPCR. (**E**) TINCR and miR-210 dual luciferase schematic diagram of the binding site and the mutant TINCR sequence. The binding sites and binding evidence are shown in Supplementary Material 1, Fig. S1E. (**F**) TINCR and miR-210 dual luciferase reporter detection relative fluorescence intensity results. (**G**) The effects of TINCR on luciferase expression after cotransfection with anti-miR-210 and control

To further study the molecular mechanism of TINCR in LSCC, we used TargetScan and the miRDB website to search for potential target miRNAs of TINCR and identified miR-210 as a potential target of TINCR (Supplementary Material 1, Fig. S1E, Supplementary Table 1, and Supplementary Table 2). miR-210 was detected in laryngeal cancer tissue samples by qPCR. As shown in Fig. 4A and B, the level of miR-210 in LCSS tissue was significantly higher than that in adjacent tissues (*P <* 0.05) and was negatively correlated with TINCR (R^2^ = 0.4847). To further study the relationship between TINCR and miR-210, the expression of miR-210 in laryngeal cancer cell lines was detected. As shown in Fig. 4C, the laryngeal cancer cell line TU212 and other laryngeal cancer cell lines highly expressed miR-210. TU212 cells expressed high levels of TINCR, and the expression of miR-210 was significantly lower than that of the parental cell line. To further understand the function of miR-210 in the laryngeal carcinoma TU212 cell line, we customized a mimic and an inhibitor of miR-210 and tested miR-210 after transfection. As shown in Fig. 4D, the miR-210 mimic was upregulated nearly 40 fold after mimic transfection. The inhibitor effectively interfered with the expression of miR-210, and compared to the control, the difference was statistically significant (*P <* 0.05).

Figure 4E shows the targeted binding sites between TINCR and miR-210, as well as the mutation sequence of TINCR for the construction of the mutant plasmid. To study further whether TINCR adsorbs miR-210 by “sponge action,” wild-type (Wt) and mutant (Mut) sequences of TINCR were inserted into the pGL3 plasmid. The resulting dual-luciferase reporter genes were cotransfected with the miR-210 plasmid into TU212 cells to detect luciferase activity. The results showed that the luciferase activity of the wild-type TINCR reporter gene was significantly inhibited by miR-210 in TU212 cells (Fig. 4F). However, luciferase activity was significantly increased in the miR-210 inhibitor group (Fig. 4G). This result confirmed that miR-210 can bind to TINCR through predicted sites.

### TINCR inhibits the proliferation and spread of cancer cells by targeting miR-210

**Fig. 5:**
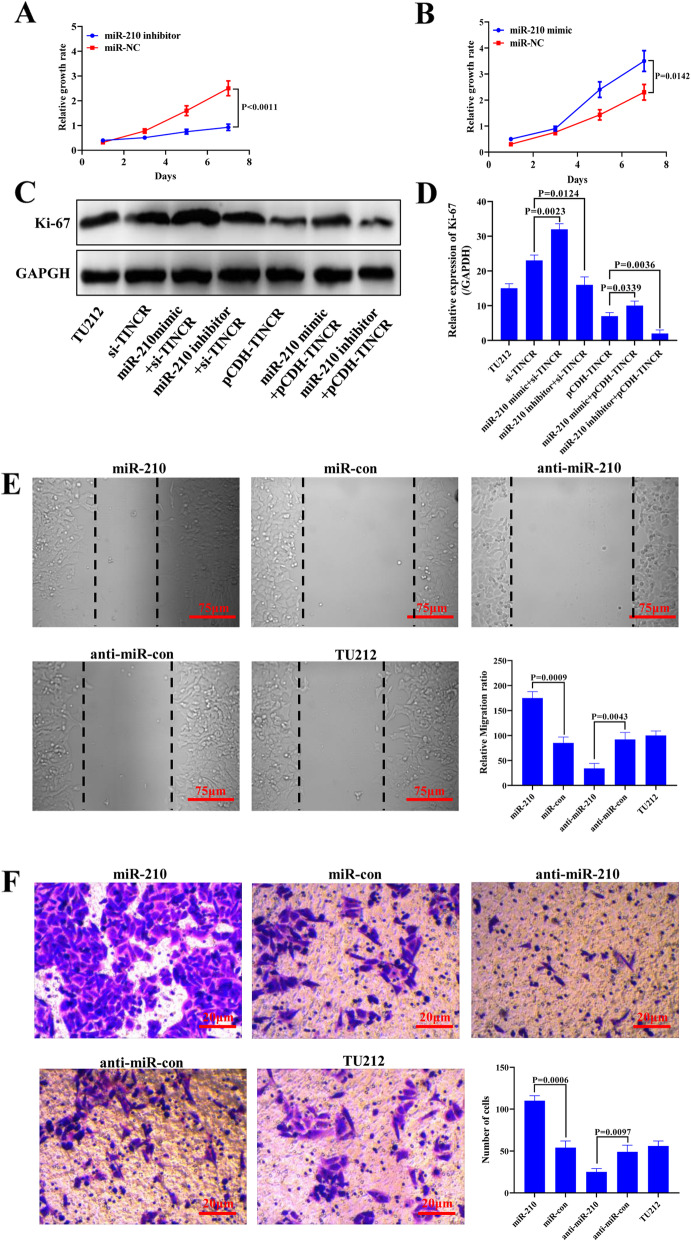
TINCR inhibits the proliferation and spread of cancer cells by targeting miR-210. (**A**) The relative cell growth rate after adding miR-210 inhibitor to TU212 cells. (**B**) Relative rate of TU212 cell proliferation after the addition of miR-210 analogues. (**C**) WB detection of the proliferation-related protein Ki67, indicating that miR-210 overexpression can reverse the inhibitory effects of TINCR on cell proliferation. (**D**) A quantitative statistical graph of WB detection results by ImageJ software. (**E**) Quantitative statistical chart for detecting cell migration distance for cell scratch experiment. (F) Quantified statistical graph of Transwell detection cell invasion results and the average number of Transwell cells. The resolution is 72 dpi

At different time points after cell transfection, CCK-8 assays were used to detect changes in cell proliferation. In the presence of anti-miR-210, the proliferation of TU212 cells was inhibited significantly (Fig. 5A). After adding the miR-210 mimic, the proliferation of TU212 cells increased (Fig. 5B). Western blot analysis showed that miR-210 upregulation restored Ki67 protein expression inhibited by TINCR in TU212 cells (Fig. 5C and D). These results indicated that TINCR inhibited the proliferation of cancer cells by targeting miR-210. After transfecting TU212 cells with miR-210-mimic and anti-miR-210, cell scratch experiments were conducted to investigate the effects of miR-210 on cell migration (Fig. 5E). The migration distance of parental TU212 cells was significantly smaller than that of TU212 cells transfected with anti-miR-con. The migration distance of TU212 cells overexpressing miR-210 was significantly larger than that of TU212 cells transfected with miR-con. Transwell experiments showed that the number of invasive cells transfected with anti-miR-210 TU212 cells was significantly lower than that of anti-miR-con transfected TU212 cells. In comparison, the number of invasive TU212 cells overexpressing miR-210 was significantly higher than that of TU212 cells transfected with miR-con (Fig. 5F).

### TINCR relieves the inhibitory effect on BTG2 by regulating miR-210, thereby inhibiting the proliferation and spread of cancer cells

**Fig. 6:**
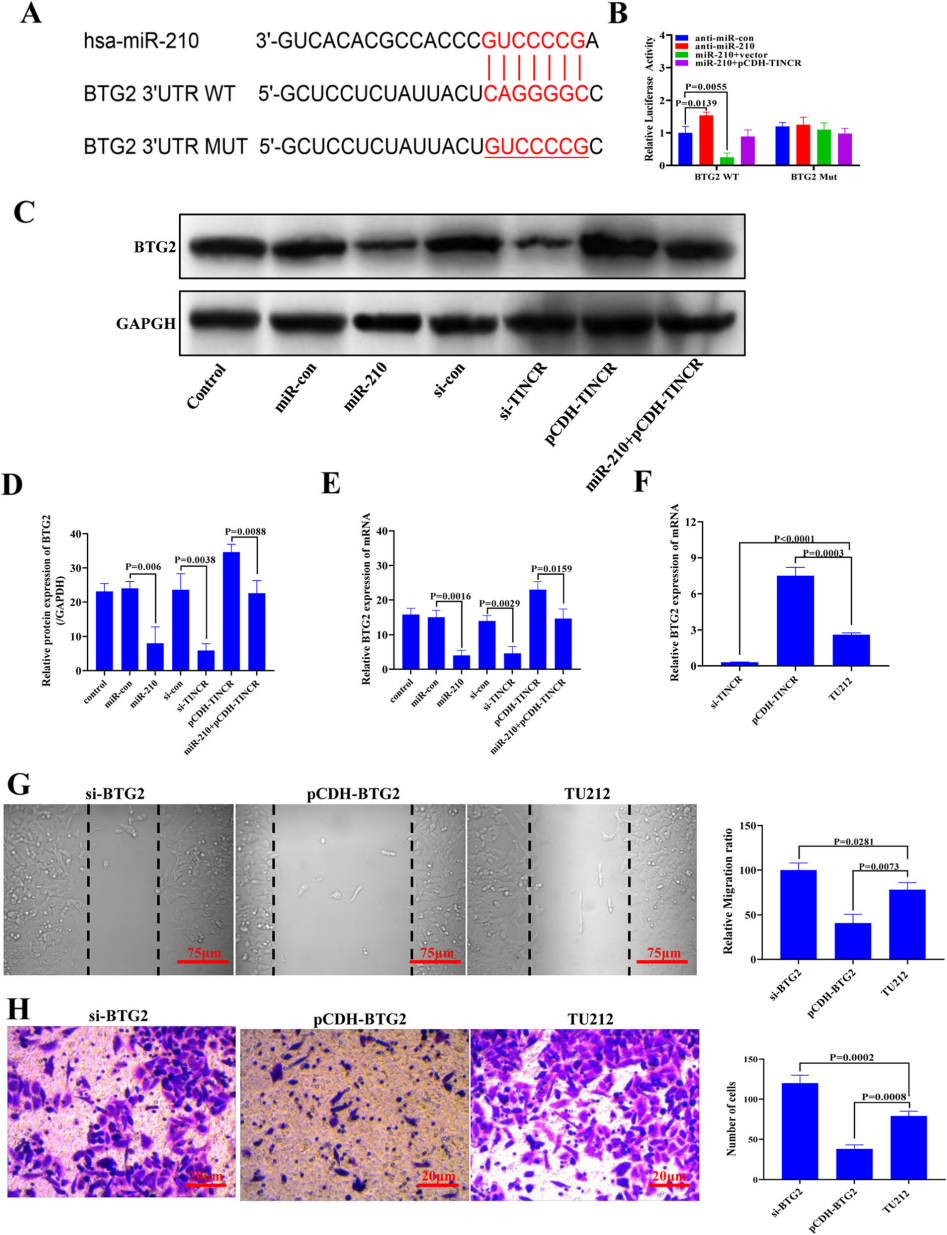
TINCR releases BTG2 inhibition by adsorbing miR-210, thereby inhibiting the proliferation and spread of cancer cells. (**A**) BTG2 and miR-210 dual-luciferase reporter binding site and the sequence of mutant BTG2. (B) Double luciferase activity reporting assay. (**C**) WB detection of BTG2 protein expression;(**D**) Statistical quantification results of gray scale of western blotting strips (**E**) The relative expression level of the BTG2 gene was detected by qPCR; (**F**) The relative expression level of the BTG2 gene in TU212 cells after transfection of the pCDH plasmid that silences or overexpresses TINCR. (**G**) Quantitative map of cell scratch results. (**H**) Quantitative statistical chart for the average number of cells detected by Transwell assays. The resolution is 72 dpi

TargetScan software was used to identify potential target mRNAs of miR-210. The results showed that miR-210 might have two complementary sites to interact with BTG2 (Supplementary Material 1, Fig. S1F, Supplementary Table 1, and Supplementary Table 2). Figure 6A shows the targeting relationship between miR-210 and BTG2 3 ‘UTR. Meanwhile, BTG2 mutation sequence was constructed according to the targeted sequence for subsequent double luciferase activity report detection. In Fig. 6B, the binding sites of miR-210-5p and BTG2 3’UTR sequences are shown with mutation sequences. To further verify this prediction, luciferase activity testing was performed after cotransfection. As shown in Fig. 6B, miR-210 upregulation significantly inhibited luciferase activity, whereas TINCR overexpression significantly inhibited miR-210. Luciferase activity inhibited the BTG2-WT reporter gene in TU212 cells. RT-PCR and Western blotting also confirmed the effect of miR-210 and TINCR on the expression of BTG2 mRNA and protein levels. Both miR-210 overexpression and TINCR knockdown significantly reduced BTG2 expression, whereas TINCR upregulation promoted the expression of BTG2 mRNA and protein levels in TU212 cells. The overexpression of TINCR significantly restored the miR-210-mediated decrease in BTG2 expression (Fig. 6C, D). RT-PCR results showed that overexpression of miR-210 or knockdown of TINCR resulted in decreased expression of BTG2 gene, with significant differences compared with control group (*P* < 0.05). However, the transfection of miR-210 could reverse the up-regulation effect of overexpressed TINCR on the expression level of BTG2 gene (Fig. 6E). Subsequently, the relative expression of BTG2 in TINCR-silenced or TINCR-overexpressing cell lines was detected by RT-PCR. The results showed that after TINCR was upregulated, the relative expression of the BTG2 gene also increased. After silencing TINCR, the relative expression of the BTG2 gene also decreased (Fig. 6F). The above results indicate that both TINCR and miR-210 can regulate the expression of BTG2, but whether TINCR reduces the growth of cancer cells by regulating the miR-210/BTG2 pathway in LSCC was unclear. Therefore, to further study the effect of BTG2 on the antiproliferative and anti-invasive effects of TINCR in LSCC cells, we conducted repair experiments. BTG2 gene knockdown reversed the inhibitory effect of TINCR overexpression on TU212 cell migration (Fig. 6G) and invasion (Fig. 6H). In contrast, BTG2 upregulation decreased the rate of TU212 cell migration (Fig. 6G) and invasion (Fig. 6H) by downregulating TINCR. These data indicated that TINCR attenuated the growth and invasion of LSCC cells by regulating the miR-210/BTG2 axis.

### TINCR overexpression can inhibit the growth of xenograft tumours in nude mice

**Fig. 7:**
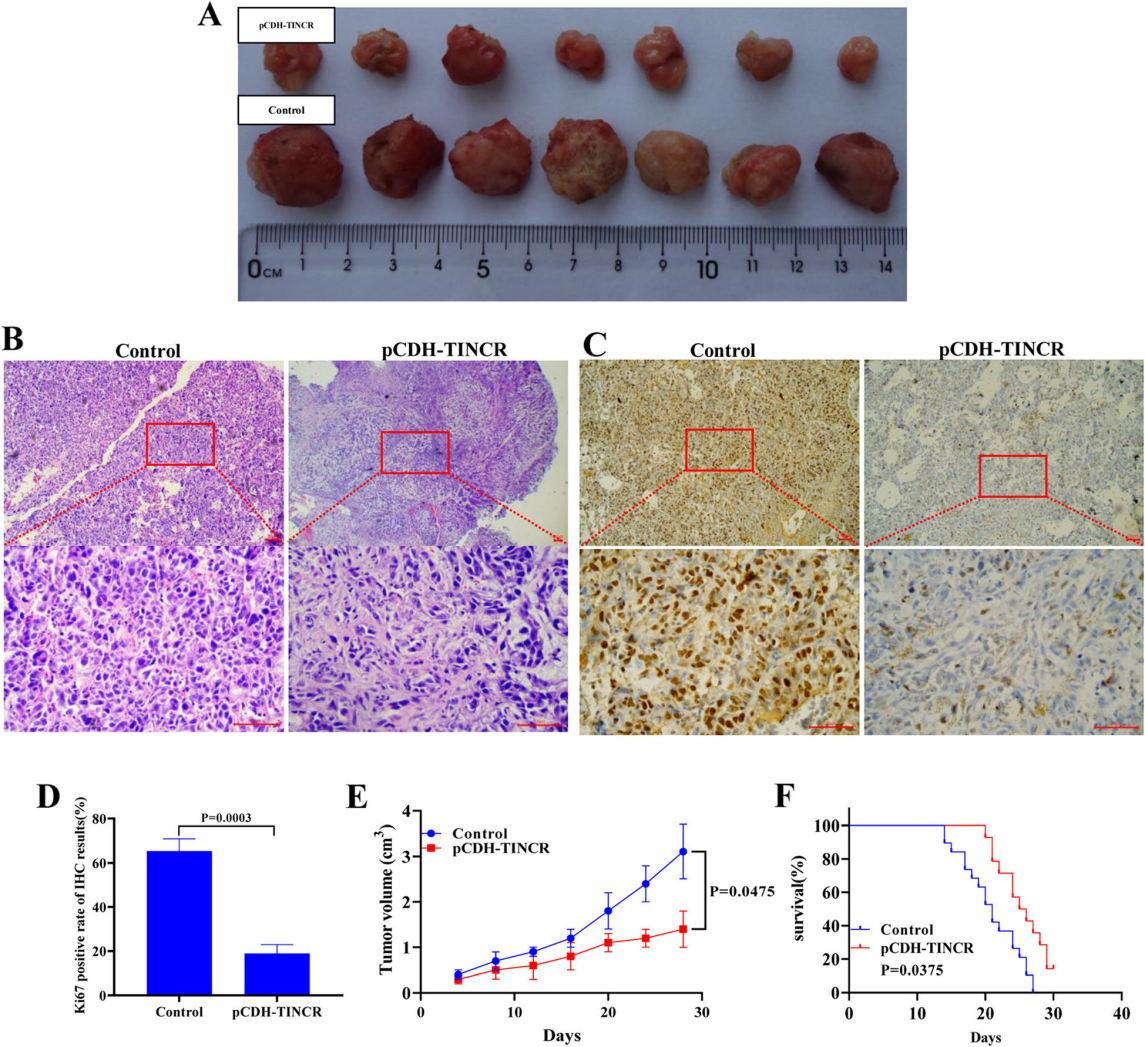
TINCR overexpression inhibits tumour development in tumour-bearing nude mice. (**A**) Tumour formation in tumour-bearing nude mice with different expression levels of TINCR. (**B**) HE staining results of tumour tissue. (**C**) Immunohistochemical detection of Ki67 protein in tumour tissue. (**D**) Histogram showing the quantitative results of C. (E) Trend of tumour volume in tumour-bearing nude mice. (**F**) Statistics of survival rate of tumour-bearing nude mice. The resolution is 96 dpi

To provide evidence for the carcinogenic role of TINCR in LSCC in vivo, we used a xenograft nude mouse model. Sixteen mice were injected subcutaneously with 1 × 10^5^ TU212 cells, and they all developed detectable tumours. As shown in Fig. 7, the xenograft growth from the cell line treated with pCDH-TINCR lentivirus was significantly inhibited compared to the control group (Fig. 7A and E). As shown in Fig. 7B, the tumour cells in the control group were denser and more compact, whereas in the TINCR-overexpressing group, the tumour construct was loose and infiltrated by more mesenchymal cells. Additional results showed that the expression of Ki67 was significantly lower in the TINCR-overexpressing group compared with the control group, indicating that tumour cell proliferation was inhibited. The survival rate of tumour-bearing mice overexpressing TINCR was significantly higher than that of control mice (Fig. 7F, *P <* 0.05). Subsequently, IHC staining for Ki67 was performed in the transplanted tumour tissue from TINCR-overexpressing TU212 cells (Fig. 7C). Tumour volumes were reduced in this group.

## Discussion

The TINCR gene is located on human chromosome 19 between *ZNRF4* and *SAFB2* and is transcribed to obtain a full-length 3.7 kb transcript [7, 16] that promotes epidermal differentiation through a post-transcriptional mechanism. Fluorescence in situ hybridization experiments showed that TINCR is enriched in the differentiation layer of human epidermal cells [7]. During epidermal differentiation, TINCR expression is increased at least 150-fold compared with the basal level. However, it is downregulated in human squamous cell carcinoma specimens, which is consistent with the decreased degree of differentiation in squamous cells. When TINCR is absent, the expression of epidermal tissue-specific genes is inhibited. The expression of 394 genes was inhibited, including FLG, LOR, ALOXE3, ALOX12B, ABCA12, CASP14, and ELOVL3. The epidermis lacking TINCR lacks terminal differentiation structures such as keratin hyaluronate particles and complete lamellar bodies. These studies suggest that TINCR plays an essential role in squamous cells, and its absence or abnormal function may lead to abnormal differentiation.

The literature has shown that TINCR may exhibit different functions in different tumours. TINCR overexpression inhibits the proliferation and metastasis of colorectal cancer cells by promoting EpCAM cleavage [8]. In a 16-year oncogene study, common epithelial squamous cell carcinomas (such as cervical cancer, head and neck cancer, and lung cancer) often exhibit ZNF750 deletion. TINCR is one of the downstream targets of ZNF750, and it mediates ZNF750 tumour suppression and the expression of important molecules that induce differentiation [17]. However, there is also evidence that in bladder cancer, TINCR promotes tumorigenesis and cancer progression by regulating cell proliferation and apoptosis [18, 19]. Silencing TINCR by small interfering RNA can significantly inhibit bladder cancer cell proliferation and migration, thereby inhibiting the further development of bladder cancer [20]. In this study, we first detected the expression level of TINCR in cancer tissues by fluorescent quantitative PCR. Compared to normal tissues, the TINCR levels were much lower in LSCC tissues. This result indicates that the involvement of TINCR in the development of LSCC is of great significance in clinical treatment. Through online analysis of prognostic data, we also found that patients with head and neck tumours with high expression of TINCR had a better prognosis. The overexpression of TINCR in the laryngeal cancer cell line TU212 significantly inhibited cancer cell proliferation, migration, and invasion, whereas TINCR knockdown promoted proliferation and invasion, which was consistent with research in bladder cancer [21]. The results showed that TINCR expression in LSCC tissue cells was significantly downregulated. TINCR overexpression significantly inhibited the proliferation and invasion of human LSCC cells, and thus we focused on analysing the role and mechanisms of TINCR in LSCC. Based on Transwell experiment results, TINCR overexpression inhibited the migration of TU212 cells by approximately 49%, and the cell invasion efficiency decreased by approximately 55.3%. Based on the above results, we believe that TINCR can inhibit the progression of LSCC by inhibiting proliferation, migration, and invasion during the development of laryngeal carcinoma.

Increasing experimental evidence shows that miR-210 plays a vital role in cancer development and has apparent clinical significance. For example, in oropharyngeal squamous cell carcinoma, miR-210 can promote tumour initiation and development by activating the ISCU [22]. In a systematic bioinformatics study, miR-210 was significantly upregulated in laryngeal carcinoma tissues. In combination with 5 other lncRNAs and 4 mRNAs, it had an essential effect on the prognosis of LSCC [23]. In this study, we found that miR-210 levels were upregulated in LSCC tissues and cell lines. In their study, Zuo et al. [24]. showed that laryngeal cancer cells were exposed to hypoxia, which resulted in increased miR-210 expression. Unfortunately, their study did not compare miR-210 expression in normal cells with cells experiencing hypoxia. In addition, in this study, differential gene expression data were collected from the RNA of 5 cell lines, and the results are thus more reliable. Consistent with the research findings, the downregulation of miR-210-5p inhibited the proliferation and invasion of LSCC cells. Conversely, the upregulation of miR-210 promoted the proliferation and invasion of LSCC cells. In addition, in Supplementary Materials 1 (Fig. S1C and Fig. S1D), we added Transwell and CCK-8 results for several cell lines with high levels of TINCR expression. The results suggested that the invasion and proliferation of laryngeal carcinoma cells with high TINCR expression were lower than those of TU212 cells with low TINCR expression. This result suggests that TINCR can inhibit the proliferation, invasion, and metastasis of LSCC cells. Low TINCR expression may indicate a low degree of malignancy for LSCC, which may be an important marker for clinical interpretation.

It has been reported that TINCR regulates the malignancy of tumours through two mechanisms. First, as a molecular sponge of microRNAs (miRNAs), it acts as a competing endogenous RNA (ceRNA) of miRNAs and regulates target mRNAs by competing with miRNAs [25, 26]. Second, TINCR directly binds to proteins in the cytoplasm, such as EpCAM, BRAF, and STAU1, thereby directly affecting proteolysis or regulating the stability of downstream target genes through interactions with proteins [27, 28]. In the past 5 years in tumour-related research, increasing evidence has shown that lncRNAs are a critical component of a ceRNA-mediated regulatory network and that they participate in tumorigenesis through interaction with miRNAs [4, 5, 12, 14]. Because the expression of miR-210 in LSCC overcomes the effects of TINCR, we speculated that TINCR acts as a miR-210 sponge to inhibit the initiation and development of LSCC. Subsequent miRNA-target programme analysis and related tests verified our inference. Additionally, the transfection of miR-210 reversed the inhibitory effects of TINCR on the proliferation and invasion of LSCC cell lines. BTG2 is a tumour suppressor gene, and the protein encoded by this gene is a member of the BTG/Tob family. This family has structurally related proteins with anti-cell proliferation properties, and the encoded protein is involved in cell cycle regulation [29–32]. Many studies have shown that regulation of the expression of BTG2 protein can effectively inhibit cancer initiation and development. A study showed that BTG2 expression is downregulated in hepatocellular carcinoma and that it suppressed stem cell-like characteristics adjacent to cancer [33]. As early as 2010, a study found that in LSCC, BTG2 was downregulated, and miR-21 could target and inhibit the expression of BTG2 and thereby inhibit its promotion of growth, proliferation, and migration [34]. Combined with the results of this study, it was inferred that the overexpression of BTG2 could block the proliferation and invasion of LSCC. Accordingly, the downregulation of BTG2 could induce the proliferation and invasion of LSCC cells, suggesting that BTG2 has a tumour-suppressive effect in LSCC. The informatics analysis combination predicted that BTG2 was the target of miR-210, and the dual luciferase reporter verified this prediction. Through the analysis of the fluorescence intensity change results, it was shown that miR-210 bound the 3′ UTR of BTG2, thus degrading the luciferase gene and reducing its expression. In addition, TINCR increased the expression of BTG2 by sponging miR-210. However, for the study of TINCR in laryngeal cancer, we only focused on miRNAs and did not determine its binding effect on proteins, which will be an important direction for further study of TINCR. In this study, we also found that the downregulation of BTG2 reversed the inhibitory effect of TINCR on the proliferation and invasion of LSCC cells. Further experiments with tumour-bearing nude mice showed that TINCR upregulation inhibited tumour growth. All the above data indicate that TINCR inhibited the progression of LSCC by regulating the miR-210/BTG2 axis. In addition to verifying TINCR function in vitro, we also established a laryngeal cancer xenograft model to investigate its effects in vivo. Consistent with the in vitro results, TINCR overexpression restricted the growth of TU212 xenografts in nude mice, and the survival of tumour-bearing mice was prolonged. We also observed the abolished expression of Ki67, suggesting that TINCR plays an essential role in the proliferation of laryngeal cancer cells.

Despite the novel findings, there are several deficiencies in this study, including the following: 1. Although in vivo experiments were included, few cell lines were studied in vitro, and the construction of multiple cell lines with stable TINCR overexpression was not performed; the results may not be reproducible in other laryngeal cancer cells. 2. Due to insufficient clinical trial data and the small number of patient samples, further sample inclusion and data collection may be performed in a larger scope to support the experimental conclusions.

## Conclusion

In conclusion, our research showed that TINCR inhibited the proliferation and invasion of LSCC cells through the TINCR/miR-210/BTG2 signalling pathway and that it participated in cell cycle regulation. TINCR could be used as a potential molecular marker and therapeutic target for LSCC.

## Supplementary Information

**Additional file 1: Supplementary Table 1.** TargetScan7.1__miR-210-5p.predicted_targets.

**Additional file 2: Supplementary Table 2.** TINCR mirna predict result1 miRDB.

**Additional file 3: Supplements Figure 1.** A Gel electrophoresis of mRNA extracted from the tissue of a patient with laryngeal squamous cell carcinoma; B The relative expression levels of lncRNA genes were detected by qPCR; C Transwell detects the invasion ability of different laryngeal squamous cell carcinoma cell lines; D CCK-8 detects the proliferation ability of other laryngeal squamous cell carcinoma cell lines; E Bioinformatics Web sites predict the targeted binding of TINCR to miRNA; F Bioinformatics Web sites predict the targeted binding of hsa-miR-210-5p to miRNA.

## Data Availability

The datasets used or/and analyzed during the current study are available from the corresponding author on reasonable request.

## Abbreviations

LSCC: Laryngeal squamous cell carcinoma
cernas: competitive endogenous RNA
DMEM: Dulbecco’s modified Eagles medium
OD: Optical density
ANOVA: Analysis of variance
RISC: RNA-induced silencing complex
Wt: Wild-type
Mut: Mutant
WB: Western blot
SCC: Squamous cell carcinoma
