# Supplementary material for: TINCR inhibits the proliferation and invasion of laryngeal squamous cell carcinoma by regulating miR-210/BTG2
Source: BMC Cancer. 2021 Jun 29;21:753. doi: 10.1186/s12885-021-08513-0 (PMC8243464; doi:10.1186/s12885-021-08513-0)

**Fig S1A. Gel electrophoresis of mRNA extracted from tissue of a patient with laryngeal squamous cell carcinoma.**


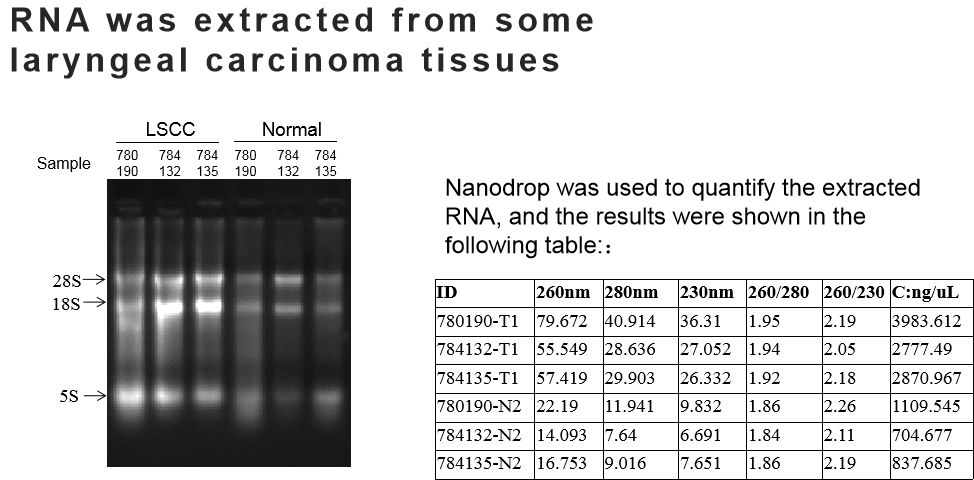


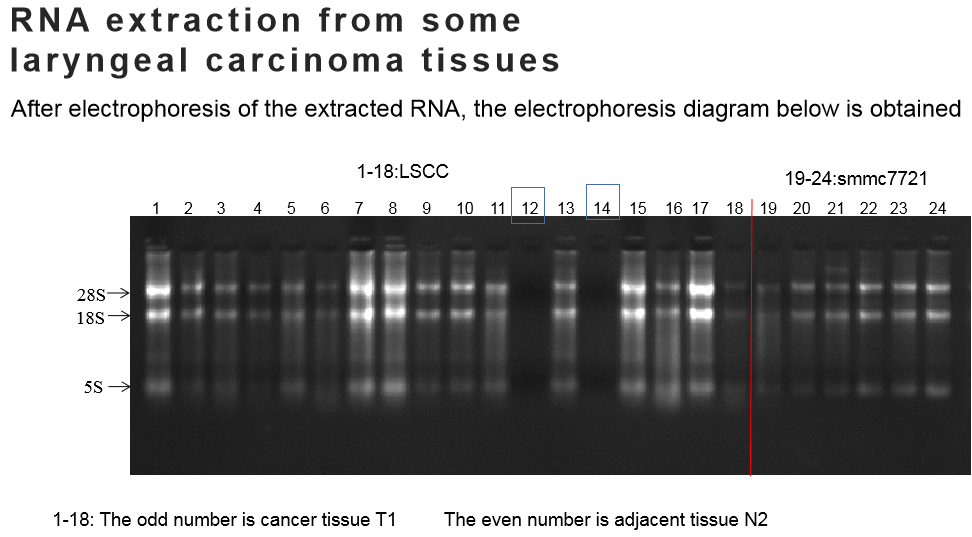


**Fig S1B. The relative expression levels of lncRNA genes were detected by qPCR.**


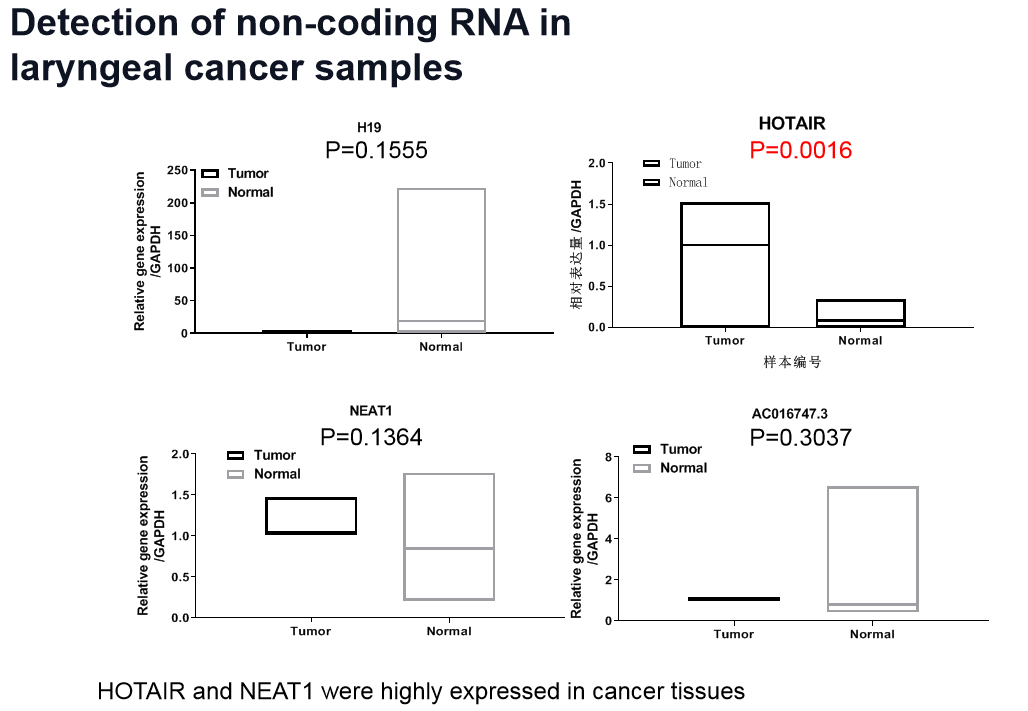


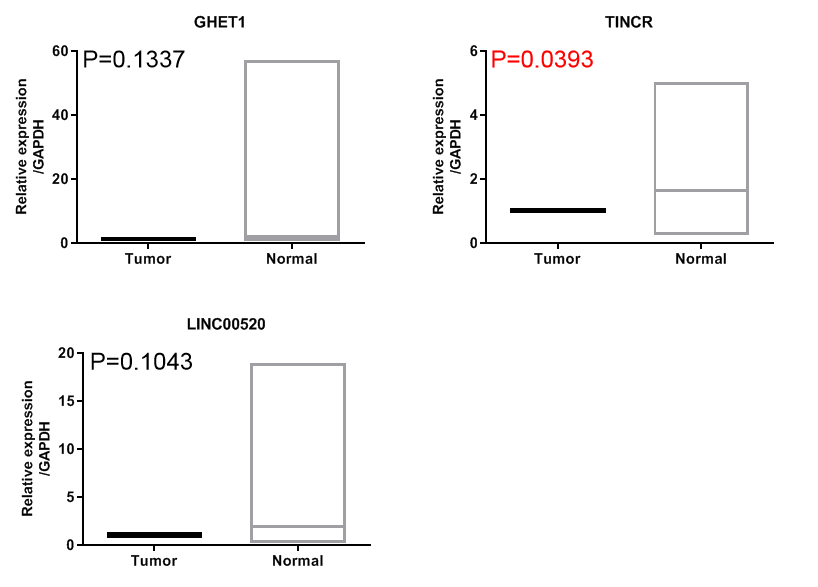


**Fig S1C. Transwell detects the invasion ability of different laryngeal squamous cell carcinoma cell lines.**

**
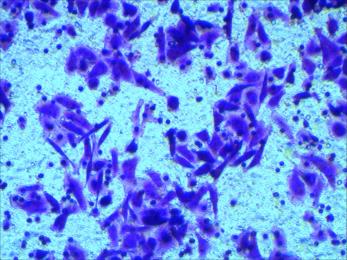

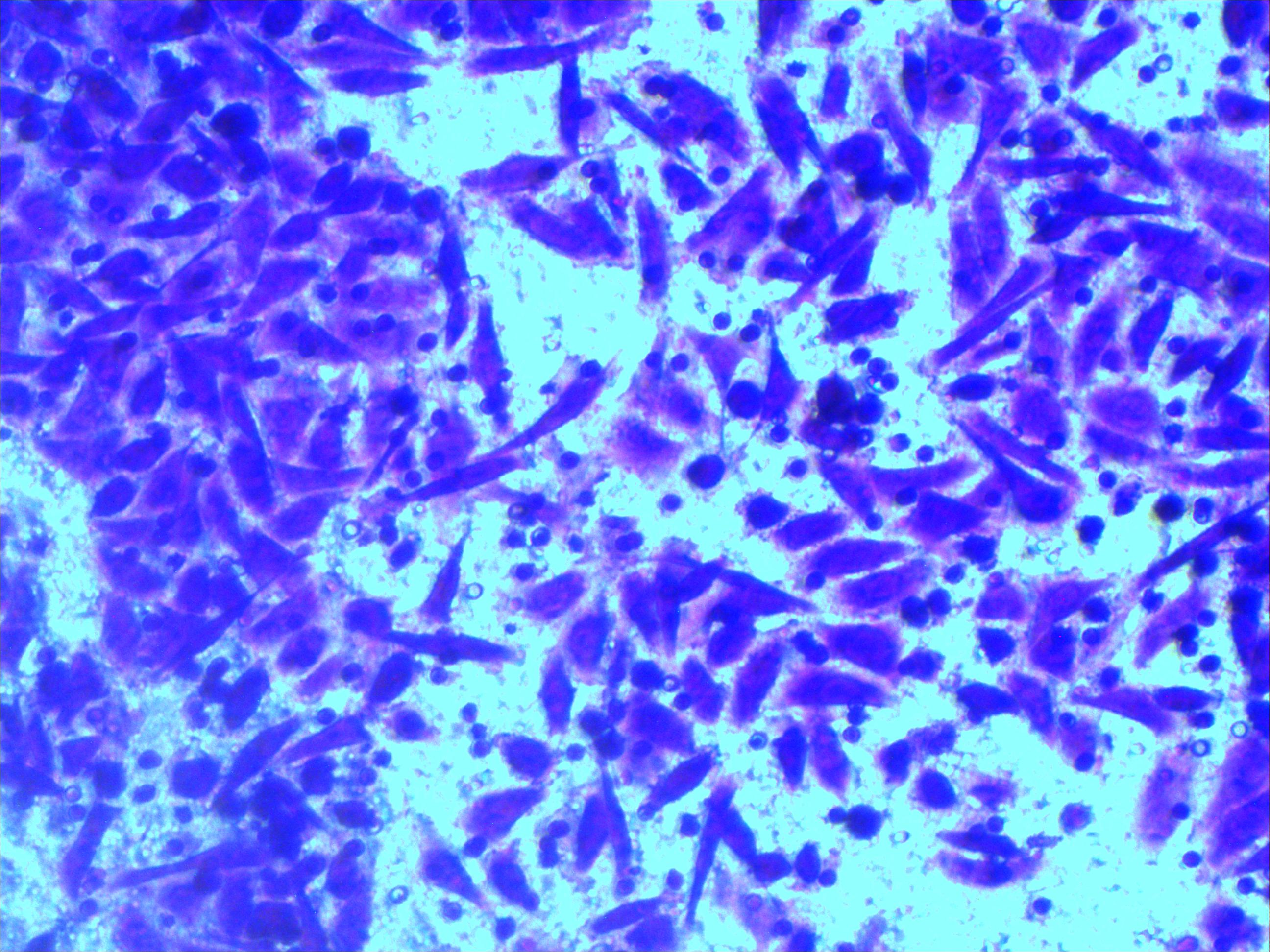

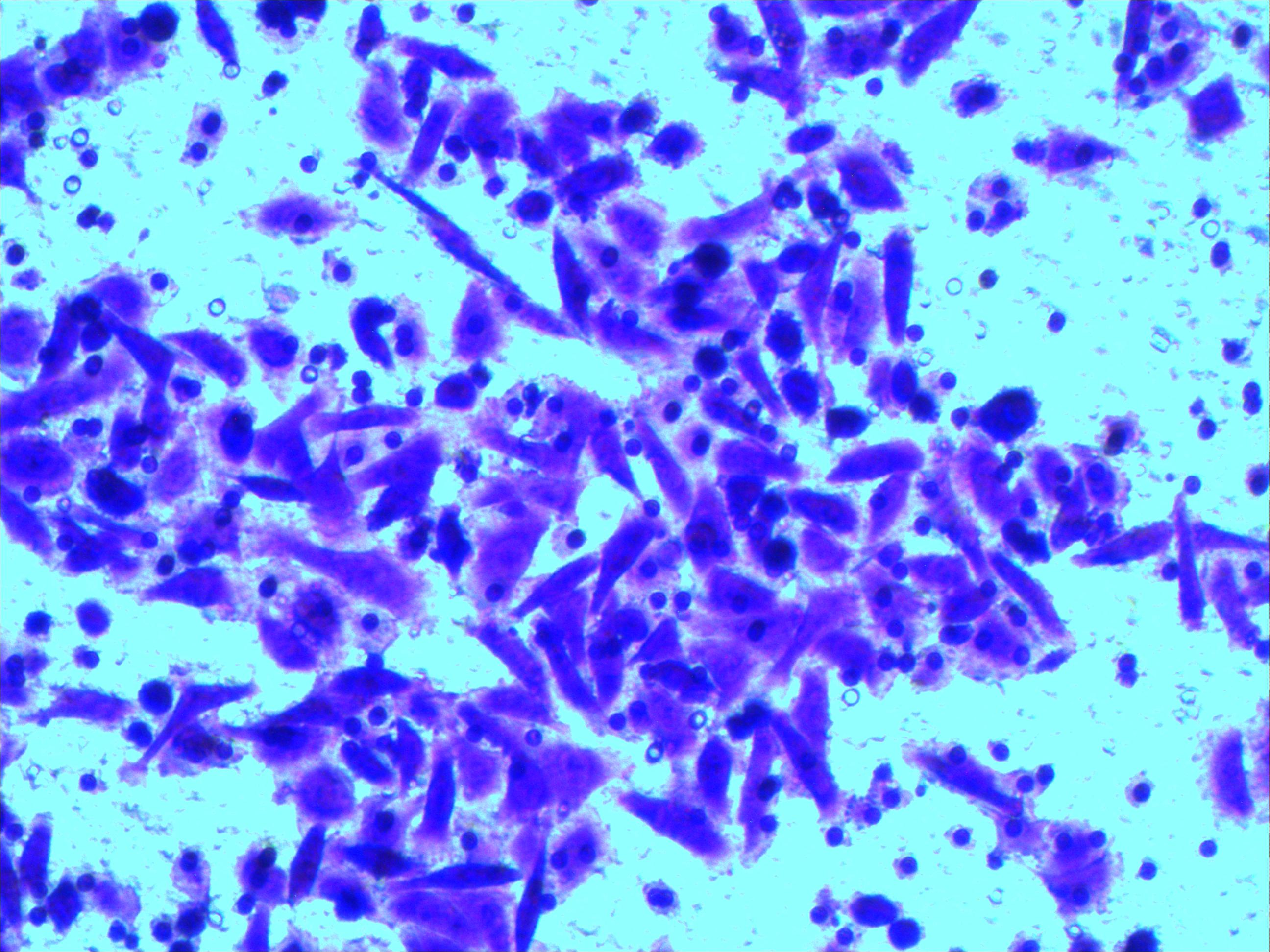
**

TU686

TU212

Hep-2

**
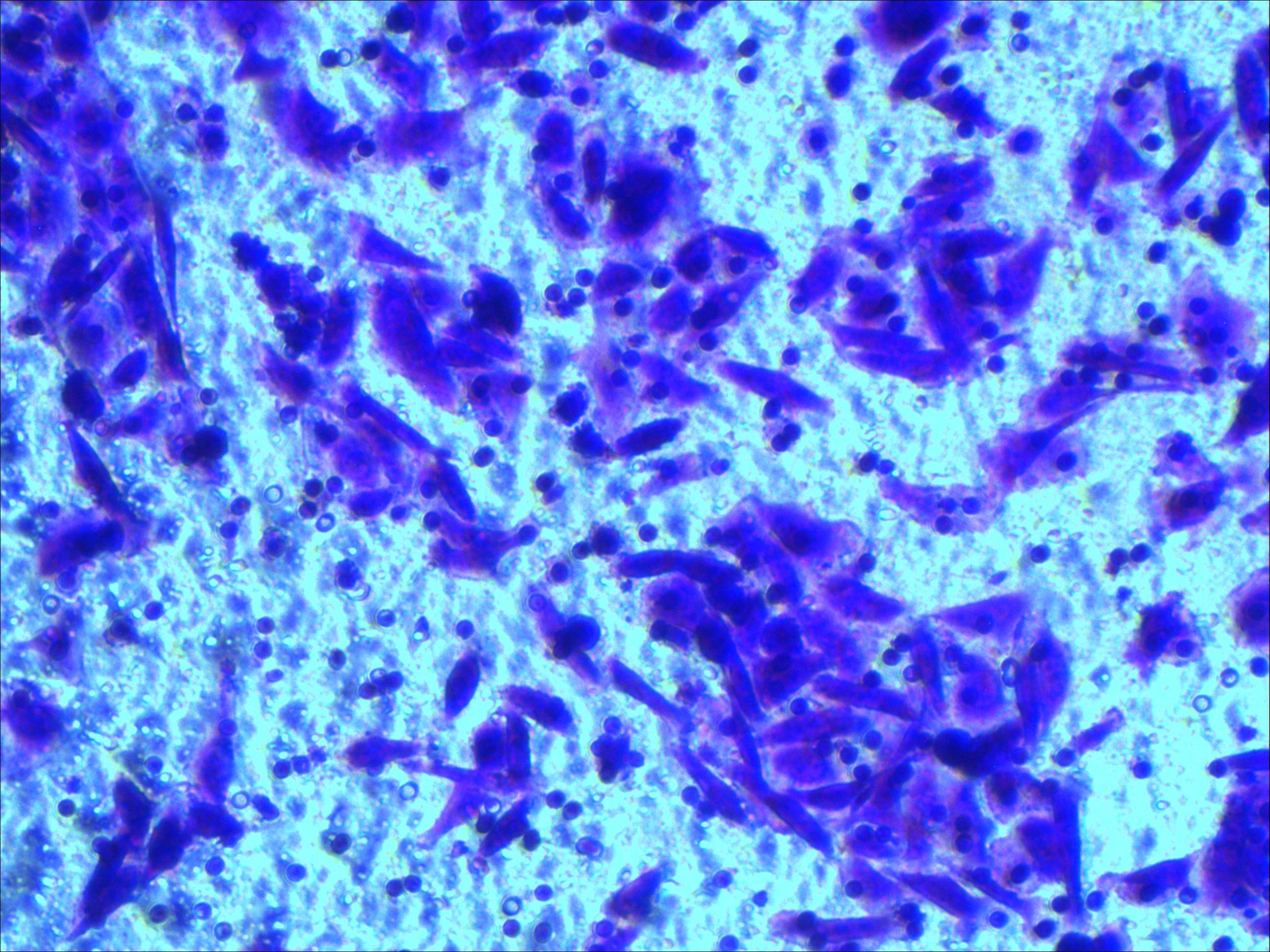

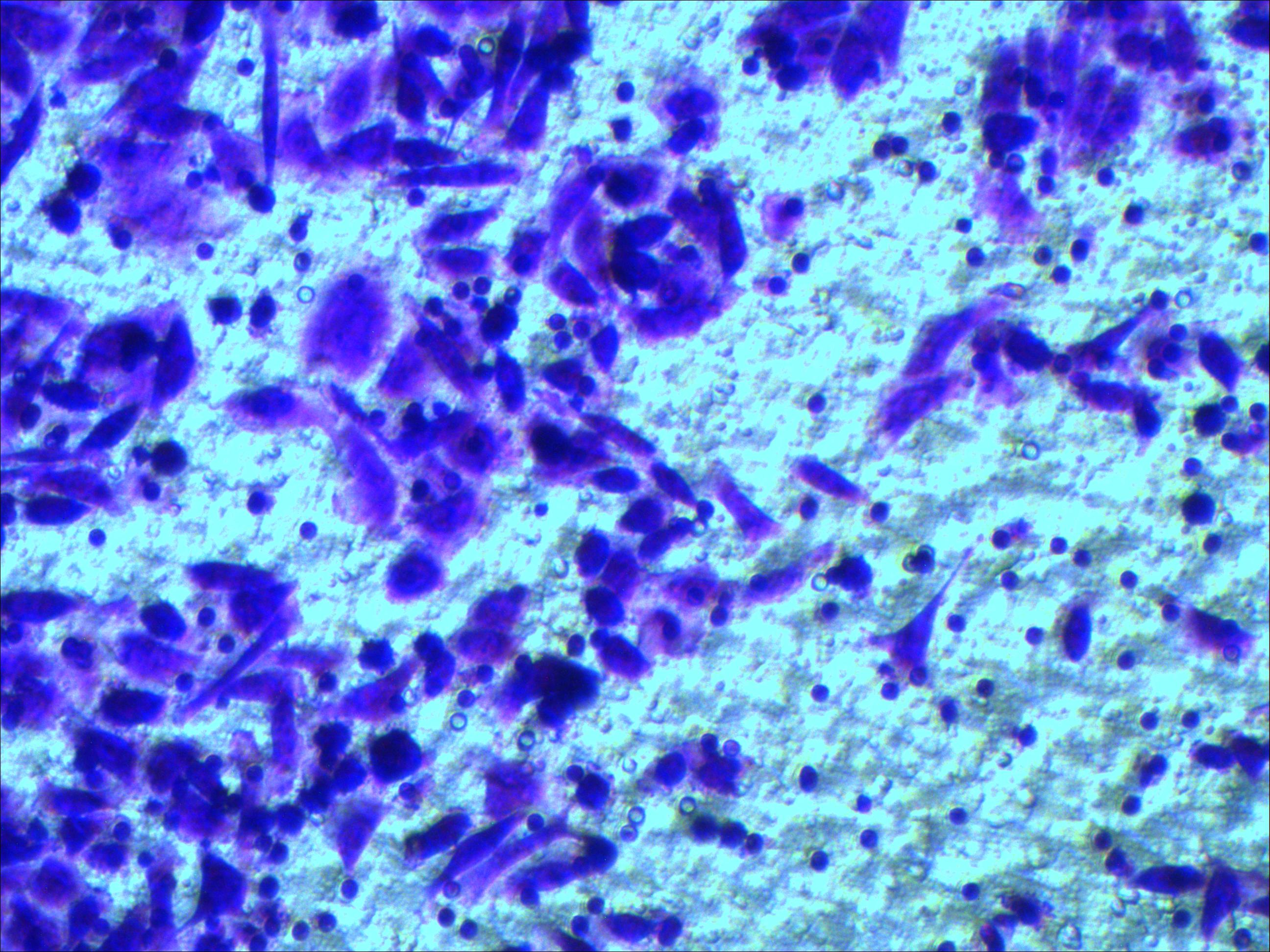
**

M4e

M2e

**Fig S1D.** **CCK-8 detects the proliferation ability of different laryngeal squamous cell carcinoma cell lines.**

**
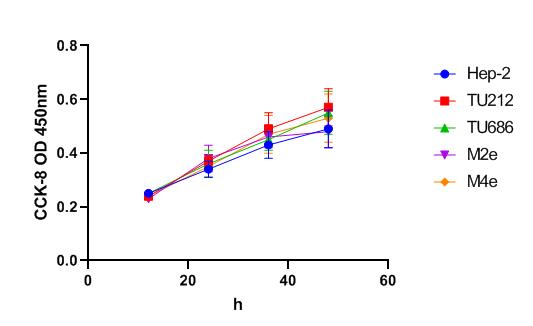
**

**Fig S1E. Bioinformatics Web sites predict the targeted binding of TINCR to miRNA.**

The miRDB online website predicted the sequences of binding sites between hsa-miR-210-5p and Tincr sequences. The sequence segments in the figure below are the nucleotide sequences of Tincr, and the blue sites are the binding nucleotide sites
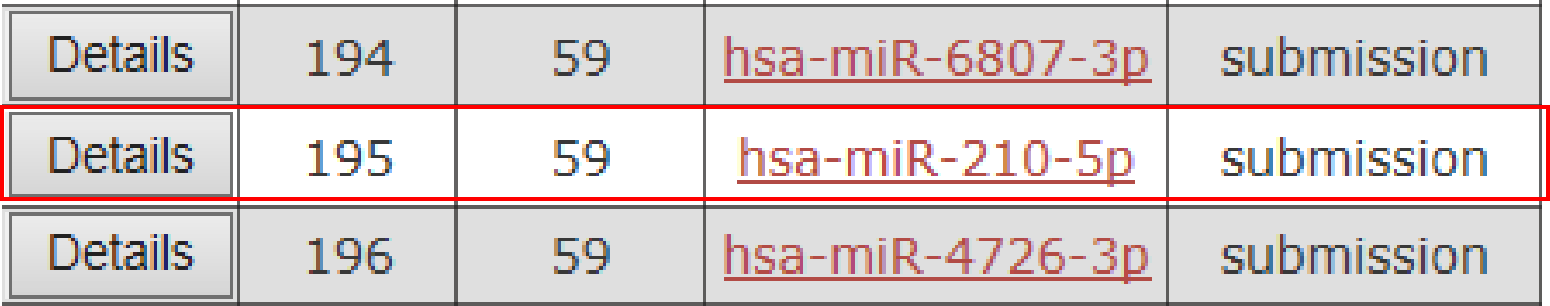


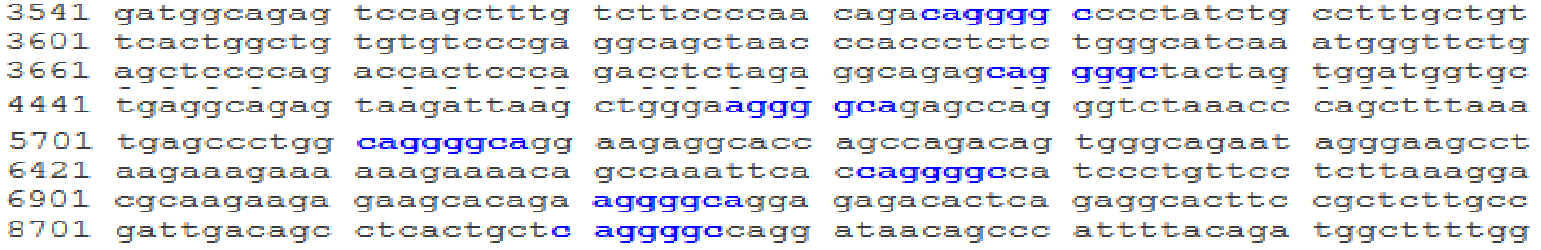


**Fig S1F. Bioinformatics Web sites predict the targeted binding of hsa-miR-210-5p to miRNA**

TargetScan predicted that hsa-miR-210-5p had targeted binding sites with 7 consecutive binding nucleotide sites in the 3’UTR sequence of BTG2.
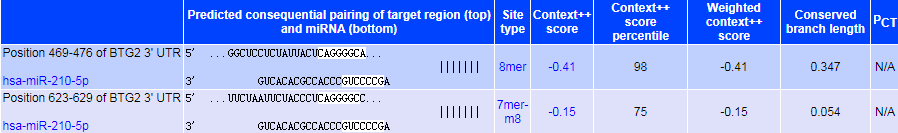

Supplement: Supplementary file 3 — Additional file 3: Supplements Figure 1. A Gel electrophoresis of mRNA extracted from the tissue of a patient with laryngeal squamous cell carcinoma; B The relative expression levels of lncRNA genes were detected by qPCR; C Transwell detects the invasion ability of different laryngeal squamous cell carcinoma cell lines; D CCK-8 detects the proliferation ability of other laryngeal squamous cell carcinoma cell lines; E Bioinformatics Web sites predict the targeted binding of TINCR to miRNA; F Bioinformatics Web sites predict the targeted binding of hsa-miR-210-5p to miRNA. [file 12885_2021_8513_MOESM3_ESM.docx]
